# Detection of rtN236T mutation associated with adefovir dipivoxil resistance in Hepatitis B infected patients with YMDD mutations in Tehran

**Published:** 2013-03

**Authors:** Seyed Hamidreza Monavari, Hossein Keyvani, Hamidreza Mollaie, Rouhollah Vahabpour Roudsari

**Affiliations:** 1 Antimicrobial Resistance Research Center, Tehran University of Medical Sciences, Tehran, Iran; 2 Department of Virology, Tehran University of Medical Sciences, Tehran, Iran

**Keywords:** Hepatitis B virus, adefovir dipivoxil, Lamivudine, resistance, mutation

## Abstract

**Background Objectives:**

The risk of adefovir dipivoxil resistance emergence has increased in lamivudine-resistant hepatitis B infected patients. The mutations known as causing adefovir resistance, rtN236T and rtA181V/T, are detected within the D and B functional domain of the HBV polymerase, respectively. In this study, we intended to determine the pre-existing adefovir-resistance mutations in patients infected with LAM resistant mutants prior to starting adefovir therapy.

**Material and Methods:**

The study included 30 patients with chronic hepatitis B with lamivudine resistance mutations in the YMDD motif that experienced viral breakthrough.

**Results:**

After alignment of protein coding sequences, the rtN236T mutation was observed in two (6.6%) patients, while twenty-eight others had neither rtN236T, nor rtA181V/T mutation. All 30 patients were infected with genotype D of hepatitis B virus.

**Conclusions:**

The early detection of LAM-resistance mutations may allow a timely chance of therapy to avoid hepatitis flare-up. This data suggests that monitoring of ADV-resistance mutations in ADV naïve patients can be considered in selecting the appropriate anti-viral regimen.

## INTRODUCTION

Chronic Hepatitis B (CHB) remains a major health problem world-wide and can lead to hepatocellular carcinoma and cirrhosis (1, 2). Several therapeutic agents including pegylated interferon and nucleotide/nucleoside analogues such as lamivudine, adefovir, telbivudin, entecavir and tenofovir (3, 4) are available.

Lamivudine has been widely used as the first-line therapy in patients with chronic hepatitis B with well-documented efficiency and safety profile (5, 6). Long term usage of lamivudine leads to development of drug resistance which is mainly associated with mutations in the YMDD motifs, substitution of methionine by either valine (rtM204I) or isoleucine (rtM204I) in the tyrosine-methionine-aspartate-aspartate (YMDD) motif of the HBV polymerase C domain (7–9). The rtL180M mutation may occur concurrently with YMDD mutations and serves as a compensation for better replication fitness (10). The risk of emerging LAM resistance is 14-32% in the first year and rises up to 70% by the fifth year (6).

Adefovir dipivoxil (ADV) is a nucleotide analogue that interferes with the reverse transcriptase activity of hepatitis B virus (HBV) polymerase upon viral replication. ADV has become an alternative treatment for HBV infection regarding the high rate of lamivudine resistance during prolonged viral therapy (11). However, viral resistance to ADV has been shown after either switching to or adding adefovir to previous regimen (ADV add-on therapy) in patients with LAM resistance (12, 13). The risk of developing adefovir resistance was 2% in the first year and 25-30% by the second and fourth years of mono-therapy in treatment-naïve cases respectively (12–14). The risk of ADV resistant emergence increased to 20% in LAM-resistant patients with switching to ADV mono-therapy within 1.5 years of ADV treatment (3). The mutations known as causing adefovir resistance, rtN236T and rtA181V/T (15, 16), are detected within the D and B functional domain of the HBV polymerase, respectively (17).

The aim of the present study was to determine the prevalence of the pre-existed ADV-resistance mutations in patients infected with LAM resistant mutants of HBV prior to starting ADV therapy.

## MATERIALS AND METHODS

Thirty chronic hepatitis B patients with the documented presence of genotypic resistance mutation to lamivudine were identified between October 2010 to December 2011. After at least 6 months lamivudine administration; the emergence of YMDD mutations was identified in those patients using restriction fragment length polymorphism (RFLP) methods (18).

### Biochemical tests and viral markers

Laboratory tests were performed at baseline and on a monthly basis during treatment. Serum ALT levels, HBeAg, anti-Hbe and HBsAg were determined using ELISA kits (Dia.Pro. Diagnostics, Italy) according to manufacturer's instructions. HBV DNA level in serum was measured using COBAS Amplicor (Roche Diagnostic, USA), this assay has a detection range between 2×10^2^-10^9^ copies/ml.

HBV DNA was extracted from 140 µl of serum using the QIAamp DNA mini kit (Qiagen Ltd. Germany) according to the manufacturer's instructions. Extracted DNA eluted in 100 µl of elution buffer and stored at -20 °C until use.

### Direct sequencing

To amplify the HBV polymerase gene by the nested PCR, we employed the primers and PCR program of Osiowy et al. (19). Briefly, the primers: spr1F (5’-GTTCAGGAACAGTAAGCCC-3’) and the reverse primer spr1R (5’- GAAAGGCCTTGTAAGTTGGCG-3’) were used in the first round PCR. The inner primers: sense, spr2F (5- GGTGGACTTCTCTCAATTTTCTAGG-3) and antisense spr2R (5- ACTTTCCAATCAATAGGCC-3) were used for nested PCR. The following PCR thermal-cycling program was performed: 35 cycles consisting of 94°C for 30s, 56 °C for 30s (first round) or 50°C for 30s (second round), and 72°C for 40s. The intended fragment were amplified using 2X PCR master mix solution (i-tag, iNtRON Biotechnology, Inc. Korea) with 5 µl of DNA extract and 2 µl of the first round PCR product. After the amplification of polymerase gene, the amplicons (730 bp) were visible after agarose gel electrophoresis and gel purified using High Pure PCR Product purification kit (Roche Diagnostic Gmbh). The purified PCR products were bi-directionally sequenced commercially (SEQLAB, Germany) using inner primers.

To perform the phylogenetic analysis, obtained sequence data were used to identify the genotype of each sample, using the NCBI genotyping tool (20). HBV polymerase encoded protein sequences were translated from each sample nucleotide sequences using Bioedit software (The BioEdit Sequence Alignment Editor software, Department of the Microbiology, North California State University) and confirmed by visual inspection. Sequences were then aligned with HBV polymerase coding sequences from the Gene Bank data base using CLUSTAL W program (http://www.ebi.ac.uk/Tools/msa/clustalw2).

## RESULTS

### Baseline characteristics

Baseline characteristics of enrolled patients prior to starting ADV therapy are listed in Table 1. All 30 patients were already infected with genotype D of hepatitis B virus. All of the patients showed viral breakthrough after at least 6 months of LAM therapy, the mean serum HBV-DNA level was 1×10^6.2^ copy/ml. HBeAg was positive in 28 (93%) of cases. The median ALT level was 177 IU/L (Table 1). LAM resistance analysis using RFLP method identified the type of YMDD mutations; 24(80%) patients had the rtM204I mutation, 2 (6.6%) had the rtM204V and 4(13.3%) had both.

**Table 1:** Baseline characteristics of patient and lamivudine resistance mutation profile.

Baseline characteristics of patient	
No. of patients	30
Age (years), median (range)	38 (20-58)
Gender (M/F)	23/7
HbeAg positivity, n (%)	28 (93%)
ALT (IU/L, median, range)	177 (35-1200)
HBV-DNA (log_10_ copies/ml), mean±SD	6.3 ± 1.33

**LAM resistance mutation profile (n, %)**	

rtM204V/I	30 (100%)
rtM204I	24 (80%)
rtM204V	2 (6.6%)
rtM204I+V	4 (13.3%)

### Detection of ADV resistance mutations by direct sequencing

**Fig. 1:**
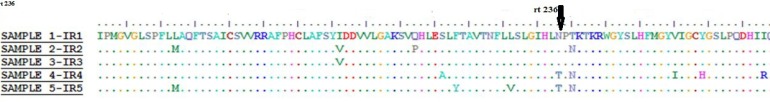
Alignment of coding sequences for detection of resistance mutations within the HBV polymerase.

After alignment of protein coding sequences, the rtN236T mutation was observed in two (6.6%) patients (Fig. 1), while twenty eight others had neither rtN236T, nor rtA181V/T mutation (Table 2.) Genetic distance was estimated using the Kimura two-parameter matrix (28) and phylogenetic tree was constructed by the neighbor-joining (NJ) method (29). Bootstrap resampling and reconstruction were carried out 1,000 times to confirm the reliability of the phylogenetic tree (Data not shown).

**Table 2:** Detection of adefovir dipivoxil resistance associated mutations within the HBV polymerase.

Genotype	No. (%) of patients		

	rt181	rt236	rt181/236
D	0 (0%)	2 (6.6%)	0 (0%)

## DISCUSSION

The emergence of resistant mutants to nucleos(t)ide analogues is the major cause of treatment failure in patients with CHB (2). LAM is the most common oral antiviral drug for the treatment of CHB. However, because of its widespread consumption, an increasing number of the emergence of LAM-resistant mutants has been reported increasingly.

Currently, the mostly accepted therapeutic guideline to overcome the LAM-resistance problem is ADV add-on treatment (11). In this study we included patients with LAM resistant mutations only before starting ADV treatment, because the aim of this study was to determine the pre-existing ADV-resistance mutations in those patients. A multivariate analysis (including adefovir treatment HBV DNA levels, ALT levels, HBeAg status) showed the baseline characteristics of the included patients.

The results of alignment of protein coding sequences indicated the rtN236T mutation was observed in two (6.6%) patients, while 28 others had neither rtN236T, nor rtA181V/T mutation.

There have been a number of studies to support the correlation between clinical response to antiviral agents and the HBV genotype, and yet there are still controversial reports (21, 22). It appears that genotype A develop LAM resistance more frequently or quite rapidly in comparison with genotype D (24). However, several reports have shown no association between genotype and the emergence of LAM resistance (25, 26).

In 2005, a report indicated an association between existence of HBV genotype D and an increased risk of ADV resistance (27). In present study since all patients were infected with HBV genotype D, the incidence of ADV resistance does not reflect true association between genotype D and the emergence of ADV resistance rate. Thus, a larger study with inclusion of other HBV genotypes is necessary to deeply understand the relationship.

Recent studies suggest new alternative strategies to conquer the rapid and frequent development of drug resistant mutant viruses. Newly approved antiviral agents which are able to suppress viral replication robustly, will probably decrease the likelihood of selecting resistance (12, 13).

Pre-existing ADV-resistance mutations such as rtN236T, prior to starting ADV mono-therapy or ADV add-on treatment may adversely affect its antiviral efficacy. Moreover the early detection of LAM-resistance mutations may allow a timely chance of therapy to avoid hepatitis flares. This study suggests that monitoring of ADV-resistance mutations in ADV naïve patients can be of a great importance.
